# *Lactobacillus paracasei* DSMZ16671 Reduces Mutans Streptococci: A Short-Term Pilot Study

**DOI:** 10.1007/s12602-013-9148-9

**Published:** 2013-09-08

**Authors:** Caterina Holz, Christiane Alexander, Christina Balcke, Margret Moré, Annegret Auinger, Maren Bauer, Lauren Junker, Jörg Grünwald, Christine Lang, Markus Pompejus

**Affiliations:** 1 ORGANOBALANCE GmbH, Gustav-Meyer-Allee 25, 13355 Berlin, Germany; 2 analyze&realize AG, Waldseeweg 6, 13467 Berlin, Germany; 3 BASF New Business GmbH, 4 Gartenweg-Z25, 67063 Ludwigshafen, Germany; 4 BASF Corporation, Fine Chemical and Biocatalysis Research, 500 White Plains Road, Tarrytown, NY 10591 USA; 5 Present Address: BASF SE, Carl-Bosch-Strasse 38, 67063 Ludwigshafen, Germany; 6 Present Address: BASF Corporation, Research Bioactive Materials, 500 White Plains Road, Tarrytown, NY 10591 USA

**Keywords:** Dental caries, Humans, Sugar-free candy, Lactobacillus pro-t-action, Co-aggregation, Saliva

## Abstract

Reducing the burden of pathogenic mutans streptococci is a goal of oral health. *Lactobacillus paracasei* DSMZ16671, even after heat-killing, specifically co-aggregates mutans streptococci in vitro and retains this activity in human saliva. In rats, it reduces mutans streptococcal colonization of teeth and caries scores. This pilot study sought to assess the potential of heat-killed *L. paracasei* DSMZ16671 (pro-t-action^®^) to reduce levels of salivary mutans streptococci in humans, using sugar-free candies as a delivery vehicle. A randomized, placebo-controlled, double-blind in vivo study of three groups examined the short-term effect of sugar-free candies containing 0 (placebo), 1, or 2 mg/candy piece of heat-killed *L. paracasei* DSMZ16671 on the levels of salivary mutans streptococci determined before and after consumption of the candies. The candies were consumed 4 times during 1.5 consecutive days. Compared to the placebo group, the test groups’ saliva had significantly reduced mutans streptococci as an immediate effect. These results suggest the use of heat-killed *L. paracasei* DSMZ16671 in suckable candies as a method to reduce mutans streptococci in the mouth and, thereby, caries risk. We think this a new concept and strategy for caries prevention and management.

## Introduction

Global increase in the prevalence of dental caries is a significant health problem, and reduced colonization of mutans streptococci is correlated with reduced risk of caries occurrence [1–3]. While there is some indication that caries is a multi-microbial disease [4], mutans streptococci are considered the predominant caries inducing microbial group with special importance in early colonization in children, and caries is associated with increased prevalence of mutans streptococci in the dental plaque biofilm adherent to teeth [5–7].

Mutans streptococci use teeth as primary colonization sites [8]. These bacteria are shed into saliva during oral function. From suspension in saliva, mutans streptococci can either re-adhere to the teeth or existing plaque biofilm or be swallowed. Prevention of their re-adhesion to the plaque by specific co-aggregation with *L. paracasei* DSMZ16671, promoting their oral clearance, could thus provide a strategy to reduce a key caries risk factor, benefiting oral health without generalized non-specific oral floral disruption [9]. The *Lactobacillus* cells co-aggregate with the mutans streptococci that are in suspension in the saliva.

*Lactobacillus paracasei* DSMZ16671 selectively co-aggregates with mutans streptococci in vitro and in saliva, but does not co-aggregate other commensal bacteria of the oral microflora [10]. In rats, *L. paracasei* DSMZ16671 inhibits *S. mutans* colonization of teeth and significantly reduces caries in rats already infected by it [9].

As a test of the hypothesis that this strategy for caries prevention can be applied to humans, we conducted a short-term, pilot, in vivo, randomized, placebo-controlled, double-blind study using sugar-free candies as the heat-killed *L. paracasei* DSMZ16671 delivery vehicle.

## Materials and Methods

### Test Products

Boiled, hard, sugar-free candies that were either supplemented or not supplemented with *L. paracasei* DSMZ16671 were produced for this study by DR.C SOLDAN^®^ GmbH (Nuremberg, Germany), a company certified to have met the requirements of International Food Standard (IFS) version 5 for the development, manufacture, and packaging of drops, fruit jelly sweets, and pastilles (category 14). The pasteurized and spray-dried *L. paracasei* DSMZ16671 had been added as the active ingredient in its commercially available form (Lactobacillus pro-t-action^®^, BASF Future Business GmbH, Ludwigshafen, Germany). The dosage of *L. paracasei* DSMZ16671 in the candies was confirmed from the sodium content of the candies as measured by atomic spectrometry. Briefly, the candies were produced by mixing, then boiling, all ingredients (except flavoring agents and *L. paracasei* DSMZ16671) until achieving a homogeneous melt. The melt was then cooled to 80 °C, whereupon flavor and *L. paracasei* DSMZ16671 were added, while mixing. This hot melt was pressed into forms, cooled, and packaged as candies. Basic ingredients for the test candies were isomaltitol, maltitol, water, sucralose, and mint oil. Test and placebo candies were identical in weight (2.5 g), size, color, and flavor.

### Study Design and Subjects

A randomized, placebo-controlled, double-blind, in vivo study was done with 3 study arms: placebo, test group 1 (1 mg *L. paracasei* DSMZ16671/candy), and test group 2 (2 mg *L. paracasei* DSMZ16671/candy). Study subjects (*n* = 60) gave informed written consent consistent with ICH Good Clinical Practice guidelines. This study was conducted at a single center in Berlin, Germany. As the trial was not a clinical trial, an ethical committee did not need to be considered, and the trial was not registered, as at the time of the trial in Germany, it was not customary for pilot-type trials to be registered.

This clinical trial was designed as a pilot to gain information regarding the treatment effect. Although there was not enough data for a sample size calculation, the trial proceeded with a sample size of 20 subjects per study group. The randomization procedure was a block randomization of 10 blocks with a block size of 6. Randomization was performed beforehand and the resulting allocation was recorded in the allocation lists. The participants those administering the treatment and those evaluating the results were blinded to the group assignment.

Subjects were excluded if under current treatment for oral or pharyngeal inflammation, restorative, gingival/periodontal, or oral surgical problems, brushed their teeth more than three times daily, used antibiotics or had dental hygiene treatment in the past 6 weeks, used full artificial dentures, were currently pregnant or lactating, had history of alcohol abuse or drug addiction, or had German language difficulties. Inclusion criteria were good health, age over 18, and written consent. There was no preselection screening of subjects for baseline levels of mutans streptococci.

### Test Schedule

The study was conducted according to the protocol and was performed over two successive days. Subjects were not to perform any oral hygiene activities or consume coffee, tea, wine, probiotic foods (such as yoghurt or cheese) from the evening prior to the first study day until completion of the study 1.5 days later. They were instructed to expectorate one mL of saliva (R-1) into a sterile 15-mL polyethylene (PE) tube, without any mechanical or taste stimulation, upon rising. This sample (R1) was to be transported to the study site within 1 h. At the study site, a second saliva sample (Pre-1) was collected before candy use. All subjects were then randomized to one of three treatment groups (placebo, test 1, or test 2) and were instructed to consume a single assigned candy piece by sucking, until fully consumed, and to refrain from chewing. The assigned candies were provided in packages coded so as to keep subjects and investigators blind. Immediately (no later than 10 min) after consuming the candy, the subjects collected another saliva sample (Post-1). Subjects who had removable partial dentures wore them throughout all study procedures.

Each subject consumed 3 more candies, one at a time, during the day, 10 min after each unsupervised meal, and was instructed not to exchange candies with other study participants.

On day 2, all subjects again collected a saliva sample upon rising (R-2), and another saliva sample (Pre-2) was collected at the study site before sucking another identically coded candy. A final saliva sample (Post-2) was collected within 10 min after consumption of this candy.

### Sampling and Sample Analysis

Except for the unsupervised saliva samples collected early in the mornings upon rising, all samples were collected on-site, under supervision by trained clinical staff. The samples were kept at room temperature and transported to the microbiology facility, and processed within a maximum of 2 h of collection, which had been established in pretests to give reliable and reproducible results (CFU). They were homogenized by sonication (15 min, ultrasonic bath Bandelin Sonorex GT120), vortexed, and a 0.5 mL volume of each was transferred into a sterile PE tube, diluted with 4.5 mL phosphate-buffered saline, and re-vortexed. One milliliter aliquot of 1:100 and 1:500 dilutions was spread in duplicate onto selective TSY20B agar [11]. The TSYB medium which was used for the detection and quantification is selective for mutans streptococci (*S. mutans, S. sobrinus,* etc.). The medium contains Bacitracin, which is an antibiotic selective for mutans streptococci among other bacteria. The plates were incubated anaerobically at 37 °C for 5 days and CFU/mL of saliva determined. Colonies were confirmed as *S. mutans* by random PCR checks using specific primers (Sm F5: AGCCATGCGCAATCAACAGGTT; Sm R4: CGCAACGCGAACATCTTGATCAG [12]). Data were recorded on Case Report Forms.

### Primary Outcome Measures

The primary outcome measure was the group change in salivary concentration of mutans streptococci immediately before (Pre-1, day 1 and Pre-2, day 2) and after (Post-1, day 1 and Post-2, day 2) consuming the test candies as compared to placebo candy. A second outcome measure was the individual study subject change in salivary mutans concentration.

### Product Acceptability Evaluation

Study subjects were asked to rate the acceptability of candies through an interview conducted by the blinded investigator.

### Statistical Methods

As mutans streptococcal counts were not normally distributed, as expected [5], all values were log-transformed to improve distribution for parametric analysis. Per protocol, data sets with less than 1,500 CFU/mL were excluded from further consideration due to the limit of detection.

Frequency distributions were analyzed using the chi-square test (*p*_chi_-value). The Kruskal–Wallis (*p*_KW_-value) method was employed to test for among-group comparisons (placebo vs. test groups). Comparisons within a group (e.g., pre- vs. post-consumption) were performed using the Wilcoxon signed-rank test (*p*_Wil_). The mean relative difference in the number of mutans streptococci before (Pre-1) and after (Post-1) candy consumption was calculated for all three groups, for day one [Post-1 vs. Pre-1] and day two [Post-2 vs. Pre-2]. Statistical analyses were performed with SPSS (SPSS for Windows, Release 19, LEAD Technologies, Inc.). Values of *p* < 0.05 were considered significant. Means were given with 95 % confidence intervals. Nonparametric analyses were performed because of the small sample size and its emphasis on the qualitative results. The *t* test was used for quantitative descriptions. CI was presented for this pilot study and represents the individual range of data and provides information about the variance of the data.

## Results

All 60 subjects were enrolled, randomized, and completed the trial. There were no statistically significant (*p* > 0.05) differences in the baseline characteristics among the sixty (60) subjects after random assignment to groups for age, gender, height, weight, BMI, heart rate, blood pressure, number of teeth, or number of smokers or vegetarians. All subjects were Caucasian. None had oral inflammatory lesions.

### Safety and Product Acceptability

No adverse events occurred in any group. For all groups, candy acceptability was rated good or very good (*p*_chi_ > 0.05).

### Range of Level of Mutans Streptococci in Baseline Samples

The majority (86.7 % on day 1 and 86.5 % on day 2) of the mutans streptococcal counts were between 10^3^ and 10^5^ CFU/mL saliva (Table 1). Counts exceeding 10^6^ CFU/mL were observed infrequently (13.3 % on day 1 and 13.5 % on day 2). Very low levels of less than 10^4^/mL were observed in 8 out of 60 test persons (13.3 %). There was no statistically significant difference between the study groups at baseline samples collected at R-1, R-2, Pre-1, and Pre-2. The ranges of mutans streptococcal levels in the samples were comparable in all groups. R-1 values—values given are log-transformed data—were 4.92 ± 0.89 for placebo, 4.95 ± 1.01 for test group 1, and 5.16 ± 0.88 for test group 2 (*p*_KW_ 0.720).

**Table 1:** Log-transformed means and 95 % confidence intervals of mutans streptococci in samples taken upon rising (*R*), before (pre) and after (post) consuming one candy, either containing 1 mg or 2 mg of *L. paracasei* DSMZ16671 or placebo

	Upon rising (*R*)	Pre-consumption (pre)	Post-consumption (post)	Absolute change (pre–post)	*p*_Wil_^#^
Day 1					
Placebo (*n* = 19)	5.16 (4.78; 5.55)	5.02 (4.63; 5.40)	4.81 (4.38; 5.24)	0.21 (0.00; 0.41)	0.053
Test group 1 (*n* = 17) (1 mg DSMZ16671)	5.36 (4.92; 5.80)	5.23 (4.82; 5.64)	5.11 (4.73; 5.49)	0.12 (−0.02; 0.26)	0.084
Test group 2 (*n* = 20) (2 mg DSMZ16671)	5.23 (4.87; 5.59)	5.16 (4.84; 5.47)	4.89 (4.52; 5.25)	0.27 (0.14; 0.41)	0.001
Day 2					
Placebo (*n* = 17)	5.11 (4.62; 5.61)	4.94 (4.46; 5.43)	4.86 (4.36; 5.36)	0.08 (−0.13; 0.30)	0.215
Test group 1 (*n* = 12) (1 mg DSMZ16671)	5.54 (5,21; 5,86)	5.43 (5.09; 5.77)	5.27 (4.93; 5.61)	0.16 (0.04; 0.28)	0.009
Test group 2 (*n* = 15) (2 mg DSMZ16671)	5.47 (5.10; 5.83)	5.43 (4.98; 5.71)	5.10 (4.73; 5.47)	0.25 (0.12; 0.37)	0.002

^#^ *p* values (Wilcoxon-rank sum test) for changes from baseline (pre- vs. post-consumption)

The samples taken on-site before consuming the candies (Pre-1 and Pre-2) were used as baseline values for the evaluation of the immediate effect of the candies on salivary level of mutans streptococci.

### Immediate Reduction in the Level of Mutans Streptococci After Candy Consumption

Table 1 shows that post-consumption salivary level of mutans streptococci compared to pre-consumption was significantly reduced in test group 2 (2 mg/piece) both on day one (*p*_Wil_ = 0.001) and on day 2 (*p*_Wil_ = 0.002). On day 2 (after day 1 consumption of 4 candies), also test group 1 (1 mg/piece) had lower salivary mutans recoveries after candy consumption compared to the pre-consumption level (*p*_Wil_ = 0.009). The level of mutans streptococci did not differ significantly in the placebo group on the 2 days of the study.

In view of the high variability in baseline counts among subjects, effects of treatments on relative changes in salivary mutans streptococcal concentration were also evaluated on a per-subject basis. On day 1, after intake of one candy, 75 % of the subjects taking placebo (ns), 65 % in test group 1 (ns), and 85 % test group 2 (*p*_Wil_ = 0.003) had reduced level of mutans streptococci. On day 2, after intake of 5 candies (four on day 1, one on day 2), only 59 % of the subjects taking placebo candies had decreased salivary mutans streptococcal counts (ns), whereas 80 % of test group 1 subjects and 75 % (both, *p*_Wil_ = 0.005) of test group 2 subjects had reduced salivary mutans streptococcal counts.

## Discussion

The results presented here as a pilot study in human adults evidence that the use of *L. paracasei* DSMZ16671, contained in an acceptable food commonly consumed, lowers salivary levels of *Strep. mutans*. This reduction is significant on two consecutive days for the higher concentration consumed and it is seen as a trend for the lower concentration candies. The data are consistent with those of Lang et al. [10] that in vitro, even when heat-killed and incubated with saliva, these bacteria cause specific co-aggregation with mutans streptococci in planktonic suspension and with those of Tanzer et al. [9] that these heat-killed bacteria when eaten with the highly cariogenic diet of rats already colonized by mutans streptococci reduce their levels on the teeth, reduce caries experience, and appear to foster clearance from the mouth. Thus, the present pilot human data support our understanding that these bacteria, even when heat-killed and contained in a sugar-free candy vehicle, foster specific co-aggregation with mutans streptococci in planktonic suspension in saliva and, thereby, an increased probability that they will be cleared from the mouth. We think it remarkable that this effect was observed after exposure to only five pieces of candy containing 1 or 2 mg of dead *L. paracasei* DSMZ16671 consumed in 1.5 days.

This double-blind, randomized, placebo-controlled in vivo study demonstrated immediate short-term reductions on salivary mutans streptococci levels through the consumption of *L. paracasei* DSMZ16671 in candies. Test subjects were not preselected according to their mutans streptococcal levels in the oral cavity. While this represented “real-life conditions,” it did probably contribute to statistical variation. Future studies are planned with larger populations with more homogeneous mutans streptococcal level subjects.

Incorporation of *L. paracasei* DSMZ16671 into sugar-free candies is but one possible form of application of pro-t-action^®^. Additionally, sugar-free candies stimulate saliva flow, a benefit to oral health [13, 14]. We could not observe any significant decrease in mutans streptococcal counts by saliva flow per se. Rather, the slight, albeit non-statistically significant reduction of salivary mutans streptococci in the placebo-candy-using group is probably attributable to the Hawthorne effect in which the mere participation in a study can change study participant behavior if subjects believe the investigators are interested in a particular outcome of such behavior, familiar in the dental clinical research literature [15–17].

Release of *L. paracasei* DSMZ16671 into saliva from sugar-free candies, leading to co-aggregation with mutans streptococci, with resultant increased probability of swallowing rather than re-attachment to the teeth or plaque biofilm [9, 10] is a plausible explanation for the reduction in the prime—albeit not sole [4]—caries pathogen in humans’ saliva.

Heat-killed *L. paracasei* DSMZ16671 has the potential to offer a method for selective reduction in mutans streptococci, thereby positively influencing the oral microbiota, without adding new or additional risk factors, as might be the case with the addition of live lactobacilli, especially those without ability to specifically co-aggregate with mutans streptococci.
